# Cell-Free Tumor DNA (ctDNA) Utility in Detection of Original Sensitizing and Resistant EGFR Mutations in Non-Small Cell Lung Cancer (NSCLC)

**DOI:** 10.3390/curroncol29020094

**Published:** 2022-02-14

**Authors:** Jason S. Agulnik, Andreas I. Papadakis, Carmela Pepe, Lama Sakr, David Small, Hangjun Wang, Goulnar Kasymjanova, Alan Spatz, Victor Cohen

**Affiliations:** 1 Peter Brojde Lung Cancer Centre, Jewish General Hospital, McGill University, Montreal, QC H3T 1E2, Canada; jason.agulnik.med@ssss.gouv.qc.ca (J.S.A.); carmela.pepe.med@ssss.gouv.qc.ca (C.P.); lama.sakr.med@ssss.gouv.qc.ca (L.S.); david.small.med@ssss.gouv.qc.ca (D.S.); victor.cohen.med@ssss.gouv.qc.ca (V.C.); 2 Lady Davis Institute for Medical Research, Jewish General Hospital, Montreal, QC H3T 1E2, Canada; andreas.papadakis.ccomtl@ssss.gouv.qc.ca; 3 Department of Pathology, Jewish General Hospital, Montreal, QC H3T 1E2, Canada; hangjun.wang.med@ssss.gouv.qc.ca; 4 OPTILAB-Montreal MUHC & Department of Laboratory Medicine, McGill University Health Center, Montreal, QC H3T 1E2, Canada; alan.spatz.med1@ssss.gouv.qc.ca

**Keywords:** NSCLC, ctDNA, EGFR mutation, liquid biopsy

## Abstract

Background: Recent studies have demonstrated the utility of cell-free tumor DNA (ctDNA) from plasma as an alternative source of genomic material for detection of sensitizing and resistance mutations in NSCLC. We hypothesized that the plasma level of ctDNA is an effective biomarker to provide a non-invasive and thus a less risky method to determine new resistance mutations and to monitor response to treatment and tumor progression in lung cancer patients. Methods: This prospective cohort study was approved and conducted at the Peter Brojde Lung Cancer Centre, Montreal. Blood was collected in STRECK tubes at four time points. DNA was extracted from plasma, and ctDNA was analyzed for the presence of mutations in the EGFR gene using the COBAS^®^ EGFR v2 qPCR (Roche) test. Results: Overall, 75 pts were enrolled in the study. In total, 23 pts were TKI-naïve, and 52 were already receiving first-line TKI treatment. ctDNA detected the original mutations (OM) in 35/75 (48%) patients. Significantly higher detection rates were observed in TKI-naïve patients compared to the TKI-treated group, 70% versus 37%, respectively (*p* = 0.012). The detection of the original mutation at the study baseline was a negative predictor of progression-free survival (PFS) and overall survival (OS). The resistance mutation (T790M) was detected in 32/74 (43%) patients. In 27/32 (84%), the T790M was detected during treatment with TKI: in 25/27 patients, T790M was detected at the time of radiologic progression, in one patient, T790M was detected before radiologic progression, and in one patient, T790M was detected four weeks after starting systemic chemotherapy post progression on TKI. At the time of progression, the detection of T790M significantly correlates with the re-appearance of OM (*p* = 0.001). Conclusion: Plasma ctDNA is a noninvasive patient-friendly test that can be used to monitor response to treatment, early progression, and detection of acquired resistant mutations. Monitoring of clearance and re-emergence of driver mutations during TKI treatment effectively identifies progression of the disease. As larger NGS panels are available for ctDNA testing, these findings may also have implications for other biomarkers. The results from ongoing and prospective studies will further determine the utility of plasma testing to diagnose, monitor for disease progression, and guide treatment decisions in NSCLC.

## 1. Introduction

One of the most exciting breakthroughs in cancer treatment is the application of personalized therapies tailored to the individual’s cancer genetic makeup. A large number of cancer genome sequencing studies collectively identified etiologic genetic changes that drive human tumor growth and progression [1,2,3]. The development of highly specific small molecules targeting these mutated proteins has provided new opportunities to tailor-fit treatments to the molecular features of the patients’ disease. These advancements of the tyrosine kinase inhibitors (TKIs), such as gefitinib and erlotinib, which target the epidermal growth factor receptor (EGFR), have led us to personalize the treatment of EGFR-mutant (EGFRm) advanced non-small cell lung cancer (NSCLC) [4,5]. 

Currently, the gold standard for biomarker analysis, including EGFR mutations, is the sequencing of DNA obtained from tissue biopsy. In an era of personalized medicine, the need for repeat biopsies to monitor the response to treatment and tumor progression has increased tremendously. In lung cancer patients, a repeat biopsy is very challenging and incurs major clinical risks. Recent studies have demonstrated the utility of circulating tumor DNA (ctDNA) from plasma as an alternative source of genomic material to detect sensitizing and acquired resistance mutations in NSCLC [6,7,8,9,10,11]. The ability to detect the T790M resistance mutation is of particular interest, since third-generation TKIs have shown favorable results in patients with this mutation [12,13,14]. 

In addition to its potential role as a detection method, ctDNA has demonstrated utility in the surveillance of progression-free survival (PFS) and overall survival (OS) in several cancers as well as in monitoring the response to treatments [15,16,17]. 

We hypothesized that liquid biopsies using ctDNA isolated from plasma are effective to provide an alternative method to repeating tumor tissue biopsies, to monitor response to treatment by detecting the original (activating and sensitizing) mutations (OM), and to monitor tumor progression in lung cancer patients by detecting the T790M acquired resistance mutation.

## 2. Materials and Methods

This prospective cohort study was conducted between 7 July 2017 and 10 January 2021 at the Peter Brojde Lung Cancer Centre, Montreal, Canada. The study protocol was reviewed and approved by the Institutional Review Board at the Jewish General Hospital. Patients with advanced/metastatic NSCLC who were found to have an EGFR mutation on tissue biopsy were enrolled for EGFR ctDNA mutation testing utilizing the COBAS^®^ EGFR v2 qPCR (Roche) test. Plasma was prepared from blood collected in STRECK™ blood collection tubes; 8 mL of whole blood. DNA was extracted from 2 mL plasma using the Cobas ctDNA Sample Preparation Kit and then analyzed for the presence of mutations in the *EGFR* (NM_005228.4) gene according to the manufacturers’ specifications (Roche Molecular Systems, Pleasanton, CA, USA). All patients provided informed consent. The study population included 2 cohorts:

- The 1st cohort participants were TKI-treated patients. These patients were currently on EGFR-TKI therapy for EGFRm metastatic NSCLC and had not yet experienced disease progression on first-line treatment. The mean time of enrollment from the start of EGFR-TKI in this cohort was 10.5 (range 3–44) months. Four longitudinal blood samples were obtained throughout the course of treatment: at the time of enrollment (baseline), at the time of 1st follow up CT scan, at the time of progression, and 1–3 months after starting second-line therapy (Table 1).

- The 2nd cohort participants were TKI-naive newly diagnosed EGFRm metastatic NSCLC. The same 4 longitudinal blood samples were obtained throughout the course of treatment, with the exception of the 1st sample, which was drawn prior to TKI treatment (baseline) (Table 1).

The results of ctDNA testing, anonymized patient information, tumor characteristics, treatment history, and outcomes were collected.

Means with standard deviation or medians with the associated 95% confidence interval (CI) for numeric data are utilized to describe demographic and clinical characteristics of the cohort. Categorical data, such as treatment patterns, are described using frequencies and proportions. Time variables (OS, PFS) are reported as medians with 95% CI using Kaplan–Meier statistics. A *p* value of 0.05 is considered significant.

IBM SPSS 20 software for Windows was used for statistical analysis.

## 3. Results

### 3.1. Patient Characteristics

**Figure 1:**
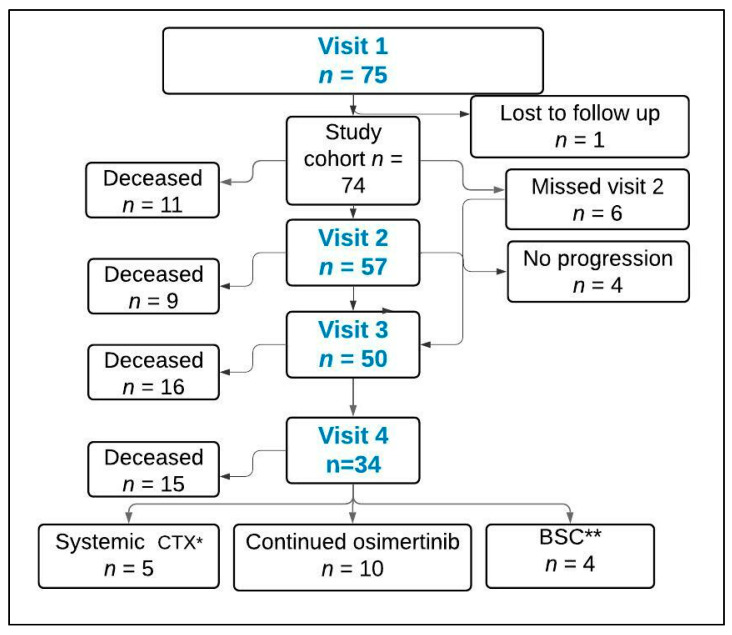
Flow chart. *—Chemotherapy, **—Best supportive care.

From January 2017 to January 2021, 75 pts were enrolled in the study. These include fifty-two patients that were receiving first-line TKI treatment (cohort 1) and twenty-three patients that were TKI naïve (cohort 2). The flowchart tracks the patient status over the four visits (Figure 1).

Table 2 summarizes the patient characteristics and response to treatment. The majority of patients were female (65%), Caucasian (63%), and non-smokers (73%). The most common EGFR mutation detected in tissue was exon 19 deletion. In context of EGFR-TKIs, the majority of patients received gefitinib. Adequate ctDNA was extracted and detected in 97% of the cases. 

### 3.2. Original Mutation (OM) Detection

**Figure 2:**
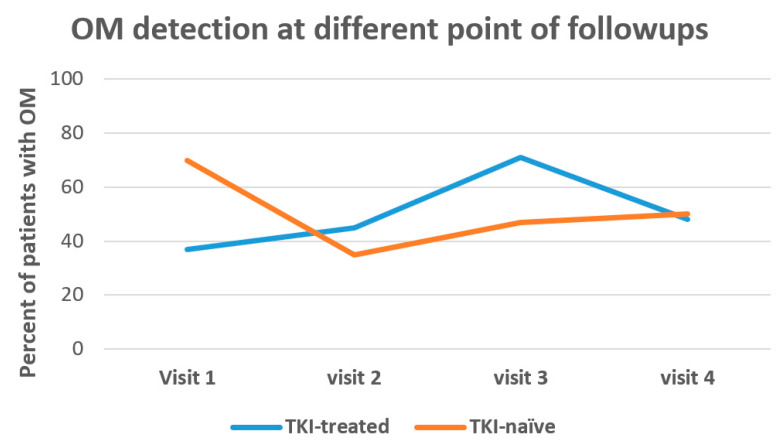
Trajectory of blood-based detection of original mutation.

ctDNA detected the OM in 35/75 (48%) patients. Significantly higher (*p* = 0.012) detection rates were observed in TKI-naïve patients compared to the TKI-treated group, 70% versus 37%, respectively (Table 3 and Figure 2). The OM detection in the TKI treated group peaked at the time of progression (Figure 2).

### 3.3. Resistant Mutation (T790M) Detection

**Figure 3:**
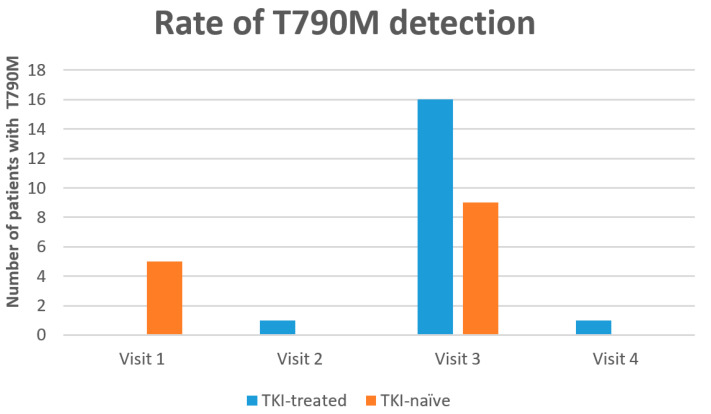
Rate of resistance mutation detection.

The resistance mutation (T790M) was detected in 32/74 (43%) patients. A de novo T790M was detected in five TKI-naïve patients: one only on ctDNA and four on both: ctDNA and diagnostic tissue sample. In 27/32 (84%) the T790M was detected during treatment with TKI: in 25/27 patients, T790M was detected at the time of radiologic progression; in one patient, T790M was detected before radiologic progression; and in one patient, T790M was detected four weeks after starting systemic chemotherapy post-progression on TKI (Figure 3). At the time of progression (visit 3), the re-appearance of OM significantly (*p* = 0.001) correlates with detection of T790M (Table 4).

### 3.4. Treatment Outcomes

**Figure 4:**
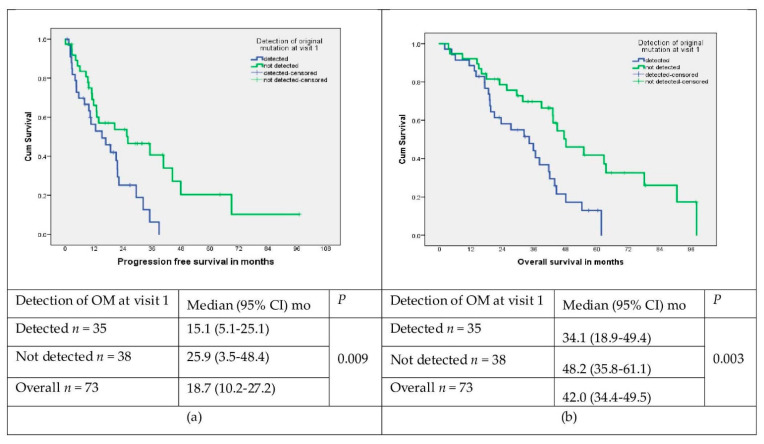
(**a**) PFS in patients with detected and not detected OM at visit 1; (**b**) overall survival in patients with detected vs. not detected OM at visit 1.

Median follow-up was 32 (range 3–98) months. At the end of the study, 51 patients were deceased. Of the 23 surviving patients: 4 continued on first line treatment, 10 continued on second line osimertinib, 5 were started on systemic therapy, and 4 were on BSC. In context of responses, 48/74 (65%) had an objective response (CR/PR) with first line TKI therapy. Disease control rate (CR/PR/SD) was 89% (66/74). The PFS on 1st line of EGFR-TKI was 18.7 (95% CI: 10.2–27.2) months, with a significantly prolonged PFS in patients with undetectable OM at the baseline (Figure 4a). Overall survival was 42.0 (95% CI: 34.4–49.5) months and was also significantly better in patients without OM at baseline (Figure 4b).

## 4. Discussion

In this prospective trial of ctDNA testing in advanced EGFRm NSCLC lung cancer, we examined the clinical utility of liquid biopsies to meet the challenges of monitoring response to targeted therapy and detection of disease progression. We found that 97% of blood samples had adequate ctDNA in plasma for testing at baseline. In comparison, it has been reported that only 80% of standard of care tissue biopsies samples had a sufficient quantity of tumor cells for genetic testing [18,19]. Agulnik et al. reported in another study that over 86% of advanced NSCLC patients enrolled had detectable cell-free DNA [12].

Our results showed that the use of serial liquid biopsies for monitoring of clearance and reemergence of EGFR activating/sensitizing mutations during TKI treatment can effectively identify the response to treatment and progression of the disease. In our study, the OM was detected in only 38% of patients being treated with EGFR-TKIs, compared to 70% in TKI-naïve patients at the baseline. This is consistent with the notion that OM is likely cleared upon TKI treatment. We have also demonstrated that the clearance of the EGFR OM at the baseline is a positive prognostic factor of response and is associated with longer PFS and OS. Patients with undetectable OM had a prolonged PFS on first-line EGFR-TKI compared to those with detected OM (26 vs. 15 months) and a better survival (48 vs. 34 months). Several other reports have demonstrated a good correlation between the detection of a driver mutation on ctDNA and treatment outcomes [13,14,20]. It has been reported that complete or partial clearance of the driver mutation was strongly predictive of a prolonged PFS and OS [13,21]. Mok et al. reported that the median PFS for patients who continued to have detectable mutant EGFR at cycle 3 was 7.2 months versus 12.0 months for patients with undetectable mutant EGFR. Similarly, median OS was also longer in patients with undetectable mutant EGFR at cycle 3 (31.9 versus 18.2 months) [21]. Other researchers measured the concentration of circulating tumor DNA and reported that rapid clearance of circulating tumor DNA concentration (ctDNA) allows us to identify patients’ therapeutic response, regardless of the type of treatment used in the first line setting [20,22]. Our results highlight the notion of Rosell et al. that “surveillance of mutations using genetic analysis of ctDNA become mandatory in the management of patients with EGFR-mutant NSCLC” [23]. 

Until recently, the detection of the T790M resistant mutation on a repeat tissue biopsy at the time of progression is considered standard of care testing. In our study, the T790M mutation detection rate in plasma was 43%. Our results are well aligned with the literature. Detection of the T790M resistance mutation ranges from 20% in REMEDY study to 63% in AURA extension and AURA2 trials [24,25,26]. It has been reported that T790M mutation detection in plasma could precede radiological progression and could potentially be used to monitor the response to first- and second-generation EGFR TKIs [24]. We demonstrate that the rate of the T790M resistance mutation detection had significantly increased compared to baseline at the time of radiological progression. The de novo T790M mutation was detected in five newly diagnosed (TKI naïve) metastatic NSCLC cases. Increasing evidence suggests that de novo T790M mutation in NSCLC patients might co-exists with EGFR activating mutations [19]. Due to the differences in detection methods and samples, the occurrence probability of de novo T790M mutation varies from 0.1% to 27% [19,27,28]. Clinical implication of this co-existing combination is yet to be investigated. In the present study, we found that, in 38% of patients failing first-line of targeted therapy, the acquired resistance T790M mutation was not detected. This is likely due to a limitation of the single-gene PCR-based testing used in this study. The investigation on the population of VALUE study by Leighl et al. showed that Guardant-360 (comprehensive NGS plasma-based test) can yield actionable or potentially actionable mutations beyond EGFR T790M in an additional 20% of patients at time of progression [29]. Detection of T790M mutation after switching to systemic treatment is likely due to tumor cells release into blood stream as an effect of chemotherapy. This event needs to be investigated further. 

Taken together, liquid biopsies show promise toward personalized medicine. Despite these encouraging results, ctDNA testing has some potential pitfalls and needs to be further investigated [18,26,30]. False negative results are the first limitation of ctDNA genotyping. It was reported that ctDNA detect T790M mutation in only 61% of positive tissue samples cases [18,19]. This might be associated with pathological tumor stage, low tumor volume, low disease burden, or clinicopathological properties that affect low ctDNA shedding [19,31,32]. The consensus is that negative results should be confirmed by a tissue biopsy [31]. To date, ctDNA is routinely analyzed by the Cobas PCR-based single gene EGFR diagnostic test. FDA approval of plasma-based NGS diagnostic panels such as Gaurdant360 and FoundationOne Liquid CDx allows us to broaden the coverage of tested genomic alteration (EGFR C797S and rare mutations, ALK, ROS1, BRAF, RET, MET, ERBB2, and PIK3CA). This initiative will lead to increased ctDNA testing as well as the availability of commercial ctDNA NGS panels for in-house testing and will provide additional evidence of the benefit of ctDNA testing. In time, these investigations, cost analysis, and real-world data will foster the foundation for ctDNA in routine clinical settings. 

Participants of our study have been enrolled before approval of osimertinib, and the majority of them received the first or second generation of EGFR-TKI. With the approval of osimertinib as a first line treatment for EGFR-mutant NSCLC, the treatment guidelines have changed accordingly.

## 5. Conclusions

Plasma ctDNA is a noninvasive patient-friendly test that can be used to monitor response to treatment, early progression, and detection of acquired resistant mutations. Monitoring of clearance and re-emergence of driver mutations during TKI treatment effectively identifies progression of the disease. As larger NGS panels are available for ctDNA testing, these findings may also have implications for other biomarkers. Results from ongoing and prospective studies will further determine the utility of plasma testing to diagnose, monitor for disease progression, and guide treatment decisions in NSCLC. 

## Figures and Tables

**Table 1:** Timing of ctDNA sampling.

Visits	Cohort 1	Cohort 2
Visit 1	Time of enrollment ^1^	Prior to any TKI treatment
Visit 2	Time of 1st follow up CT scan ^2^	Time of 1st follow up CT scan ^2^
Visit 3	Time of progression	Time of progression
Visit 4	1–3 months after starting second line therapy	1–3 months after starting second line therapy

^1^ Mean time of enrollment was 10.5 (range 3–44) months from the time of start of EGFR TKI. ^2^ Mean time if 1st CT scan was 12 (range 4–15) weeks.

**Table 2:** Patient characteristics.

Characteristics	TKI-Treated *n* (%) *n* = 52	TKI-Naïve *n* (%) *n* = 23	*p* Value	Total *n* (%) *n* = 75
Gender:				
Male	17 (33)	8 (35)	0.86	26 (35)
Female	35 (67)	15 (65)		49 (65)
Ethnicity:				
Caucasian	34 (65)	13 (56)	0.68	47 (63)
Asian	19 (35)	9 (44)		28 (37)
Smoking history:				
Ex/current smokers	14 (27)	6 (26)	0.94	20 (27)
Non-smokers	38 (73)	17 (74)		55 (73)
EGFR alterations:				
Exon 19 deletion	31 (60)	15 (65)		46 (62)
Exon 21 (L858R)	19 (36)	8 (35)	0.64	27 (36)
Exon 21 (L681Q)	1 (2)	0 (0)		1 (1)
Exon 18	1 (2)	0 (0)		1 (1)
First line TKI:				
gefitinib	43 (82)	16 (70)		59 (80)
afatinib	6 (12)	3 (13)	0.78	9 (12)
erlotinib	3 (6)	0 (0)		3 (4)
osimertinib	0	3 (13)		3 (4)
unknown (lost to follow up)	0	1 (4)		1
Best response to 1st line treatment:				
Complete response (CR)	4 (8)	2 (9)		6 (8)
Partial response (PR)	29 (56)	13 (57)		42 (56)
Stable disease (SD)	12 (23)	6 (26)	0.75	18 (24)
Mixed response	1 (2)	1 (4)		2 (3)
Progressive disease (PD)	6 (11)	0 (0)		6 (8)
Unknown (lost to follow up)	0	1 (4)		1 (1)
Adequacy of DNA in plasma				
Adequate quantity	51 (98)	22 (96)	0.99	73 (97)
Undetectable quantity:	1 (2)	1 (4)		2 (3)

**Table 3:** ctDNA detection of OM in TKI-treated and TKI-naive patients at visit 1.

Variable	TKI-Treated *n* = 50 *	TKI-Naïve *n* = 23	Total *n* = 73	Pearson Chi-Square
OM ** detected *n*(%)	19 (38)	16 (70)	35 (48)	0.012
OM not detected *n*(%)	31 (62)	7 (30)	38 (52)	
Total	50 (100)	23 (100)	73 (100)	

* In 2 cases of the TKI-treated cohort the DNA was undetectable. ** Original mutation.

**Table 4:** Correlation of detection of OM and T790M mutation.

Time	OM Detection	T790M Detection		Total	*p* Value
		Detected	Not Detected		
Visit1 ^a^	Detected	5	30	73	n/a
	Not detected	0	38		
Visit 2	Detected	1	23	57	n/a
	Not detected	0	33		
Visit 3	Detected	24	12	50	0.001
	Not detected	1	13		
Visit 4	Detected	8	7	31	n/a
	Not detected	0	16		

^a^—In 2 cases the DNA was undetectable.

## Data Availability

The data presented in this study are available on request from the corresponding author.

## References

[1] Mouliere F., Messaoudi S.E., Pang D., Dritschilo A., Thierry A.R. (2014). Multi-marker analysis of circulating cell-free DNA toward personalized medicine for colorectal cancer. Mol. Oncol..

[2] Luo H., Li H., Hu Z., Wu H., Liu C., Li Y., Zhang X., Lin P., Hou Q., Ding G. (2016). Noninvasive diagnosis and monitoring of mutations by deep sequencing of circulating tumor DNA in esophageal squamous cell carcinoma. Biochem. Biophys. Res. Commun..

[3] Messaoudi S.E., Mouliere F., Manoir S.D., Bascoul-Mollevi C., Gillet B., Nouaille M., Fiess C., Crapez E., Bibeau F., Theillet C. (2016). Circulating DNA as a strong multi-marker prognostic tool for metastatic colorectal cancer patient management care. Clin. Cancer Res..

[4] Mok T.S., Wu Y.L., Thongprasert S., Yang C.H., Chu D.T., Saijo N., Sunpaweravong P., Han B., Margono B., Ichinose Y. (2009). Gefitinib or carboplatin-paclitaxel in pulmonary adenocarcinoma. N. Engl. J. Med..

[5] Zhou C., Wu Y.-L., Chen G., Feng J., Liu X.-Q., Wang C., Zhang S., Wang J., Zhou S., Ren S. (2011). Erlotinib versus chemotherapy as first-line treatment for patients with advanced EGFR mutation-positive non-small-cell lung cancer (OPTIMAL, CTONG-0802): A multicentre, open-label, randomised, phase 3 study. Lancet Oncol..

[6] Connolly I.D., Li Y., Gephart M.H., Nagpal S. (2016). The “Liquid Biopsy”: The Role of Circulating DNA and RNA in Central Nervous System Tumors. Curr. Neurol. Neurosci. Rep..

[7] Diaz L.A.J., Bardelli A. (2014). Liquid biopsies: Genotyping circulating tumor DNA. J. Clin. Oncol..

[8] Zhang Z., Ramnath N., Nagrath S. (2015). Current Status of CTCs as Liquid Biopsy in Lung Cancer and Future Directions. Front. Oncol..

[9] Lin C.-C., Huang W.-L., Wei F., Su W.-C., Wong D.T. (2015). Emerging platforms using liquid biopsy to detect EGFR mutations in lung cancer. Expert Rev. Mol. Diagn..

[10] Maheswaran S., Sequist L.V., Nagrath S., Ulkus L., Brannigan B., Collura C.V., Inserra E., Diederichs S., Iafrate A.J., Bell D.W. (2008). Detection of mutations in EGFR in circulating lung-cancer cells. N. Engl. J. Med..

[11] Sacher A.G., Paweletz C., Dahlberg S.E., Alden R.S., O’Connell A., Feeney N., Mach S.L., Jänne P.A., Oxnard G.R. (2016). Validation of Rapid Plasma Genotyping for the Detection of EGFR and KRAS Mutations in Advanced Lung Cancer. JAMA Oncol..

[12] Agulnik J., Law J.H., Juergens R., Laskin J., Laurie S., Hao D., Ezeife D.A., Le L.W., Kiedrowski L.A., Lanman R.B. (2020). Defining VALUE: Routine liquid biopsy in NSCLC diagnosis—A Canadian trial in progress in AACR. Philadelphia.

[13] Alegre E., Fusco J.P., Restituto P., Salas-Benito D., Rodríguez-Ruiz M.E., Andueza M.P., Pajares M.J., Patiño-García A., Pio R., Lozano M.D. (2016). Total and mutated EGFR quantification in cell-free DNA from non-small cell lung cancer patients detects tumor heterogeneity and presents prognostic value. Tumour. Biol..

[14] Mayo-de-Las-Casas C., Garzón Ibáñez M., Jordana-Ariza N., García-Peláez B., Balada-Bel A., Villatoro S., Malapelle U., Karachaliou N., Troncone G., Rosell R. (2018). An update on liquid biopsy analysis for diagnostic and monitoring applications in non-small cell lung cancer. Expert Rev. Mol. Diagn..

[15] Krebs M.G., Sloane R., Priest L., Lancashire L., Hou J.-M., Greystoke A., Ward T.H., Ferraldeschi R., Hughes A., Clack G. (2011). Evaluation and Prognostic Significance of Circulating Tumor Cells in Patients With Non–Small-Cell Lung Cancer. J. Clin. Oncol..

[16] Bauernhofer T., Zenahlik S., Hofmann G., Balic M., Resel M., Pirchmoser R., Regitnig P., Ambros P., Dandachi N., Samonigg H. (2005). Association of disease progression and poor overall survival with detection of circulating tumor cells in peripheral blood of patients with metastatic breast cancer. Oncol. Rep..

[17] Hao T.B., Shi W., Shen X.J., Qi J., Wu X.H., Wu Y., Tang Y.Y., Ju S.Q. (2014). Circulating cell-free DNA in serum as a biomarker for diagnosis and prognostic prediction of colorectal cancer. Br. J. Cancer.

[18] Aggarwal C., Thompson J.C., Black T.A., Katz S.I., Fan R., Yee S.S., Chien A., Evans T.L., Bauml J.M., Alley E.W. (2019). Clinical Implications of Plasma-Based Genotyping With the Delivery of Personalized Therapy in Metastatic Non–Small Cell Lung Cancer. JAMA Oncol..

[19] Leighl N.B., Page R.D., Raymond V.M., Daniel D.B., Divers S.G., Reckamp K.L., Villalona-Calero M.A., Dix D., Odegaard J.I., Lanman R.B. (2019). Clinical Utility of Comprehensive Cell-free DNA Analysis to Identify Genomic Biomarkers in Patients with Newly Diagnosed Metastatic Non–small Cell Lung Cancer. Clin. Cancer Res..

[20] Wei Z., Wang W., Shu Z., Zhou X., Zhang Y. (2017). Correlation Between Circulating Tumor DNA Levels and Response to Tyrosine Kinase Inhibitors (TKI) Treatment in Non-Small Cell Lung Cancer. Med Sci. Monit..

[21] Mok T., Wu Y.-L., Lee J.S., Yu C.-J., Sriuranpong V., Sandoval-Tan J., Ladrera G., Thongprasert S., Srimuninnimit V., Liao M. (2015). Detection and Dynamic Changes of EGFR Mutations from Circulating Tumor DNA as a Predictor of Survival Outcomes in NSCLC Patients Treated with First-line Intercalated Erlotinib and Chemotherapy. Clin. Cancer Res..

[22] Jovelet C., Madic J., Remon J., Honoré A., Girard R., Rouleau E., André B., Besse B., Droniou M., Lacroix L. (2017). Crystal digital droplet PCR for detection and quantification of circulating EGFR sensitizing and resistance mutations in advanced non-small cell lung cancer. PLoS ONE.

[23] Rosell R., Karachaliou N. (2016). Lung cancer: Using ctDNA to track EGFR and KRAS mutations in advanced-stage disease. Nat. Rev. Clin. Oncol..

[24] Usui K., Yokoyama T., Naka G., Ishida H., Kishi K., Uemura K., Ohashi Y., Kunitoh H. (2019). Plasma ctDNA monitoring during epidermal growth factor receptor (EGFR)-tyrosine kinase inhibitor treatment in patients with EGFR-mutant non-small cell lung cancer (JP-CLEAR trial). Jpn. J. Clin. Oncol..

[25] Takahama T., Azuma K., Shimokawa M., Takeda M., Ishii H., Kato T., Saito H., Daga H., Tsuboguchi Y., Okamoto I. (2020). Plasma screening for the T790M mutation of EGFR and phase 2 study of osimertinib efficacy in plasma T790M–positive non–small cell lung cancer: West Japan Oncology Group 8815L/LPS study. Cancer.

[26] Jenkins S., Yang J.C.-H., Jänne P.A., Thress K.S., Yu K., Hodge R., Weston S., Dearden S., Patel S., Cantarini M. (2017). EGFR Mutation Analysis for Prospective Patient Selection in Two Phase II Registration Studies of Osimertinib. J. Thorac. Oncol..

[27] Wang X., Li X., Guo H., Zhu L., Peng Z., Wang J., Yang F., Guo Y. (2020). Highly Sensitive Droplet Digital PCR Method for Detection of de novo EGFR T790M Mutation in Patients with Non-Small Cell Lung Cancer. OncoTargets Ther..

[28] Wang X., Zhong D. (2019). Advanced Research on Non-small Cell Lung Cancer with De Novo T790M Mutation. Zhongguo Fei Ai Za Zhi.

[29] Laurie S., Agulnik J., Hao D., Juergens R., Ezeife D., Law J., Le L., Kiedrowski L., Shepherd F., Cohen V. (2020). 1195P The value of detecting resistance through liquid biopsy. Ann. Oncol..

[30] Cescon D.W., Bratman S.V., Chan S.M., Siu L.L. (2020). Circulating tumor DNA and liquid biopsy in oncology. Nat. Cancer.

[31] Jenkins S., Yang J.C.-H., Ramalingam S.S., Yu K., Patel S., Weston S., Hodge R., Cantarini M., Jänne P.A., Mitsudomi T. (2017). Plasma ctDNA Analysis for Detection of the EGFR T790M Mutation in Patients with Advanced Non–Small Cell Lung Cancer. J. Thorac. Oncol..

[32] Cho M.-S., Park C.H., Lee S., Park H.S. (2020). Clinicopathological parameters for circulating tumor DNA shedding in surgically resected non-small cell lung cancer with EGFR or KRAS mutation. PLoS ONE.

